# Doppler–Scintigraphy Combination with Thyroxine Profiling Enhances Diagnostic Accuracy of Thyroid Lesions: A 144-Patient Cross-Sectional Study

**DOI:** 10.3390/jcm15093364

**Published:** 2026-04-28

**Authors:** Reham Mohamed Taha, Moawia Gameraddin, Yasir Hassan Elhassan, Awadia Gareeballah, Osama Musa, Fatimah Ahmed Daghas, Ali Ibrahim Aamry, Nisreen Haj, Tasneem S. A. Elmahdi, Sahar A. Mustafa, Abdullah Fahad A. Alshamrani, Amel F. H Alzain, Awatif M. Omer

**Affiliations:** 1 Radiology Department, Alghad College for Applied Medical Sciences, Riyadh 13629, Saudi Arabia; rmohamed@gc.edu.sa (R.M.T.); nhaj@gc.edu.sa (N.H.); smustafa@gc.edu.sa (S.A.M.); 2 Department of Diagnostic Radiology, College of Applied Medical Sciences, Taibah University, Al-Madinah 42353, Saudi Arabia; awadhia1978@gmail.com (A.G.); telmahdi@taibahu.edu.sa (T.S.A.E.); ashamrani@taibahu.edu.sa (A.F.A.A.); afzain@taibahu.edu.sa (A.F.H.A.); awatefomer222@hotmail.com (A.M.O.); 3 Department of Basic Medical Sciences, College of Medicine, Taibah University, Al-Madinah 42353, Saudi Arabia; yasiranatomy2@hotmail.com; 4 Radiological Sciences Program, Batterjee Medical College, Jeddah 21442, Saudi Arabia; osamaomer9256@yahoo.com; 5 Nuclear Medicine Department, King Saud Medical City, Riyadh 11196, Saudi Arabia; f.daghas@ksmc.med.sa (F.A.D.); a.aamry@ksmc.med.sa (A.I.A.)

**Keywords:** thyroid nodules, vascularity, thyroid uptake, thyroxine, scintigraphy

## Abstract

**Background**: The characterization of thyroid lesions is essential in clinical practice. Recent advances in imaging modalities, including nuclear imaging (NM), color Doppler ultrasonography, and sonography, have markedly improved the diagnostic accuracy for thyroid nodules. **Objective**: To assess thyroid diseases using Doppler ultrasound, nuclear scintigraphy, and sonography. **Results**: In this cross-sectional single-center study, 144 patients were examined to determine their thyroid structure and function using a multimodal imaging approach. Fine-needle aspiration cytology (FNAC) indicated that most thyroid nodules were benign (62.5%), with 37.5% being malignant. Doppler vascularity demonstrated a sensitivity of 70.4% and a specificity of 40% (AUC = 0.514) for malignancy detection, while scintigraphy uptake in hypofunctioning nodules (nodules with decreased radionuclide uptake) showed a sensitivity of 37% and a specificity of 54.4% (AUC = 0.388). Thyroxine hormone levels showed a sensitivity of 57.4% and a specificity of 45.6% (AUC = 0.503) for detecting malignant thyroid nodules. In multivariate logistic regression, increased Doppler vascularity remained an independent predictor of malignancy (OR = 2.39; 95% CI: 1.15–4.96; *p* = 0.019), whereas decreased scintigraphic uptake showed a borderline effect (OR = 1.82; *p* = 0.069); high T4 level and increased uptake were not significant predictors. The combined Doppler ultrasound, scintigraphy, and thyroxine level model yielded an AUC of 0.72 (95% CI: 0.63–0.81), markedly higher than any single parameter. At the optimal Youden threshold (0.43), the model achieved 79.6% sensitivity, 68.2% specificity, and 72.4% accuracy, highlighting the superior diagnostic performance of the integrated approach for pre-FNAC stratification of thyroid malignancies. There was a strong, significant linear association between thyroxine levels and thyroid scintigraphy uptake (*p*-value < 0.001). Most patients with normal thyroxine levels exhibited decreased uptake (66.1%), whereas a minority (6.5%) demonstrated elevated uptake levels. This study found a strong correlation between mixed-echogenicity nodules and thyroid scintigraphy uptake (*p*-value = 0.019). Mixed-echogenicity nodules were most often associated with reduced uptake (57.8%), and hypoechoic nodules often had normal uptake (57.1%). **Conclusions**: The complementary integration of color Doppler vascularity, Tc-99m thyroid scintigraphy, and serum thyroxine levels yields superior Doppler–scintigraphy uptake correlation, increases the overall diagnostic accuracy, and offers a practical, non-invasive algorithm for differentiating benign from malignant thyroid nodules prior to FNAC or surgery.

## 1. Introduction

Thyroid dysfunction should be studied in many populations in order to obtain an integrated understanding of metabolic processes. It requires a delicate balance in patient care and therefore needs to be accurately diagnosed and properly managed. Management of this chronic disease has entered a new era as a result of advanced imaging techniques that have enhanced the clinical assessment of the thyroid gland in terms of its structure and physiology. Notably, Doppler ultrasound and thyroid gland scintigraphy have become vital diagnostic procedures.

The thyroid gland, located in the neck’s anterior aspect, is crescent-shaped with two fused lobes connected by a central isthmus. Due to its proximity to major structures, surgery and clinical interventions are challenging. Imaging methods like Doppler ultrasound aid in assessing blood flow [1,2,3,4].

The complex vascularized structure of the thyroid not only supports its metabolic activity, but also facilitates its surgical management. The radiological assessment of the thyroid gland requires the identification of its morphological and vascular patterns as such characteristics can influence the physiology and clinical appearance of different thyroid diseases [5,6]. This knowledge of anatomical aspects increases the precision of diagnosing and treating thyroid diseases and states, highlighting the necessity of advanced imaging techniques to evaluate structural and functional lesions [4,6].

Doppler ultrasound is a safe imaging technique for classifying thyroid disorders. In contrast, a thyroid scan uses Tc-99m or iodine-123 to assess thyroid tissue function and detect unsuspected hyperactive nodules or dysfunctional regions [7,8,9]. The integration of both imaging modalities enhances diagnostic accuracy. For example, while scintigraphy can identify hyperfunctioning nodules, Doppler ultrasound can provide insights into the vascular characteristics of these nodules, which may influence decision-making regarding interventions such as fine-needle aspiration cytology (FNAC) [10]. Furthermore, Doppler ultrasound is effective for evaluating the hemodynamic parameters of thyroid arteries, which can aid in distinguishing between different thyroid pathologies [11].

On the other hand, evaluating thyroid nodules by assessing their echogenicity on ultrasound and uptake on thyroid scintigraphy is critical for determining their functional status and potential malignancy [12]. Both imaging modalities provide complementary information that enhances diagnostic accuracy and informs clinical management strategies.

Despite the known value of these imaging modalities, there is limited research on their combined use, which could markedly improve diagnostic accuracy and therapeutic management. A previous study demonstrated that Doppler ultrasound can not only assess vascular patterns, but can also be used to monitor disease changes, such as changes in the level of autoimmunity in autoimmune thyroiditis, which can be measured using internalizing thyroid antibodies [13]. Additionally, the capacity of Doppler ultrasound to profile hemodynamics adds value to the data obtained from scintigraphy, providing a better assessment of the patient.

Thyroxine (T4) is a critical hormone produced by the thyroid gland that plays a vital role in regulating metabolism and overall thyroid function. Understanding the relationship between thyroxine levels and thyroid scintigraphy uptake is essential for diagnosing and managing thyroid disorders. Thyroid scintigraphy, a nuclear medicine imaging (NM) technique, uses radioactive isotopes such as Tc-99m or iodine-123 (I-123) to assess the thyroid gland’s functional status [14]. The uptake patterns of these isotopes provide valuable insights into thyroid activity, which is closely linked to serum thyroxine levels [14]. The clinical linking of thyroxine concentrations to scintigraphic results will greatly improve the differentiation between hyper- and hypothyroid states, which will affect treatment outcomes [15].

While ultrasound, Doppler imaging, and thyroid scintigraphy are useful for assessing thyroid lesions, most studies have focused on evaluating these techniques individually or evaluating their malignancy predictive value. The current study shifts the perspective by combining hemodynamic (Doppler vascularity), functional (scintigraphic uptake), and biochemical (serum thyroxine level) assessments into one framework. This study not only evaluated the individual diagnostic value of each modality but also considered the mechanisms involved and the extent to which the combination of these modalities enhances clinical assessments. This framework creates a practical approach to structure–function correlation that can help clarify pre-FNAC decisions and minimize invasive procedures. Therefore, this study aimed to assess the combined efficacy of Doppler vascularity, thyroid scintigraphy uptake, and serum thyroxine level as a single evaluative framework in the evaluation of thyroid lesions, which was compared to FNAC, the current gold standard.

## 2. Materials and Methods

This retrospective cross-sectional single-center study was conducted at a hospital in Saudi Arabia. All patients with thyroid symptoms were included in this study. Patients who had previously undergone thyroid surgery were excluded from this study, as were those with insufficient imaging data.

### 2.1. Data Collection

The data were collected from the Nuclear Medicine and Doppler Imaging departments’ Picture and Communication System (PACS) (Carestream Health, Rochester, NY, USA), and included patients of different ages and genders, and with different thyroid abnormalities. All patients had been assessed using sonography, Doppler imaging, and thyroid scintigraphy, and their thyroxine hormone level had been measured. A total of 144 patients with thyroid disorders treated from December 2020 to April 2022 were retrospectively studied.

### 2.2. Sonographic Procedure

A thyroid ultrasound had been performed as part of routine evaluation using a high-frequency linear array transducer (7–10 MHz) of a GE logic E9 ultrasound machine GE Healthcare, Chicago, IL, USA). The patient was positioned supine with the neck extended and a pillow placed under the shoulders. Images of each lobe and the isthmus were acquired in both the sagittal and transverse planes, followed by a calculation of the thyroid volume. Additionally, thyroid vascularity was assessed using color Doppler ultrasonography. Experts in the field confirmed the final imaging findings.

### 2.3. Tc-99m Scintigraphy

Subsequently, the patients were referred for thyroid uptake analysis and scans as part of routine clinical care. Data from thyroid scintigraphy using Tc-99m were collected retrospectively from the Nuclear Medicine Department and the PACS. Scintigraphic images and statistically significant uptakes obtained as part of standard clinical practice were reviewed and analyzed, and used to calculate the thyroid uptake percentage using the following equation:

$$Uptake\;\%=\frac{Neck\;counts-Thigh\;Counts}{(Admin.\;counts\times decay\;factor)-Background\;Counts}\times 100$$

The standard range for thyroid radiotracer uptake is 0.5% to 2%. Excessive uptake of radioactive iodine by the thyroid gland indicates hyperthyroidism, whereas insufficient uptake suggests hypothyroidism or disruption of uptake. The nodules were classified as hyperfunctioning (increased uptake), hypofunctioning (decreased uptake), or normal.

The total serum T4 concentration was quantified by radioimmunoassay in all patients.

### 2.4. Fine-Needle Aspiration Cytology (FNAC)

Fine-needle aspiration cytology (FNAC) was performed on thyroid nodules as part of the routine clinical evaluation for cytological characterization. Samples were processed according to the standard protocols and were characterized as benign, malignant, or indeterminate based on cytopathological criteria. For diagnostic performance assessment, receiver operating characteristic (ROC) curve analyses were conducted to assess the sensitivity, specificity, and area under the curve (AUC) of the Doppler vascularity and thyroid scintigraphy uptake data in differentiating benign from malignant lesions, which were compared to FNAC as the gold standard.

### 2.5. Statistical Analysis

The data were analyzed using the Statistical Package for the Social Sciences (SPSS), version 23 (IBM Corp., Armonk, NY, USA). Qualitative data were analyzed using the chi-square test. Descriptive variables were presented as means, frequencies, and percentages. A binary logistic regression model was applied to assess Doppler vascularity and its independent association with thyroid malignancy, as well as its combination with thyroid scintigraphy uptake and serum thyroxine levels. The regression model was adjusted for covariates such as benign vs. malignant, age, and sex. From the regression model, the predicted probabilities were calculated and used to calculate a combined ROC curve to evaluate the overall diagnostic performance of the model. Adjusted odds ratios (ORs) with 95% confidence intervals (CIs) were determined. A *p*-value less than 0.05 was considered significant.

## 3. Results

One hundred and forty-four consecutive patients that met the study criteria were included in the final analysis. We collected a full set of data for each participant, including gray-scale ultrasound, color Doppler flow studies, Tc-99m thyroid scintigraphy, serum thyroxine levels, and FNAC for certain nodules. The cohort consisted mostly of women (77.1%) and included participants of all ages, which made it possible to study how demographics affected the imaging and biochemical results. The largest age groups were the 41–50 and 31–41 groups (Table 1). This age distribution indicates potential variation in how thyroid disorders manifest and their prevalence in the different age groups in this sample.

Graves’ disease was the most common NM finding (41.7%), followed by multinodular goiters (16.7%) and nodules (9.7%). The diversity of lesion types emphasizes the role of NM in assessing thyroid disorders (Table 2).

There was a statistically significant association between thyroxine levels and scintigraphy uptake (*p* < 0.001), indicating a strong association between biochemical function and imaging findings (Table 3).

There was a significant relationship between scintigraphy uptake and Doppler vascularity (*p* = 0.005), suggesting that increased vascularity is generally correlated with higher functional activity (Table 4).

Doppler vascularity exhibited high sensitivity (70.4%) but lower specificity (40%) compared to scintigraphy uptake, which showed a sensitivity of 37% and a specificity of 54.4% (Table 5). The sensitivity of thyroxine hormone for detecting thyroid nodules was also higher than that of scintigraphic uptake (57.4 vs. 37.5). These findings indicate that Doppler vascularity is a more sensitive tool for malignancy risk stratification than scintigraphy uptake and thyroxine hormone level, which have moderate specificity.

Multivariate logistic regression analysis was applied to assess the combined ability of Doppler vascularity, thyroid scintigraphy uptake, and serum thyroxine levels to predict thyroid malignancies (Table 6). Increased Doppler vascularity was associated with thyroid malignancy (OR = 2.39, 95% CI: 1.15–4.96, *p* = 0.019). Less uptake in scintigraphy was considered borderline and demonstrated a weak association (*p* = 0.069). The adjusted model found that increased scintigraphy uptake and thyroxine levels were not significant predictors (*p*-values > 0.05). The model highlights the significant diagnostic value of the combined structural–functional–biochemical (S-F-B) approach.

**Figure 1:**
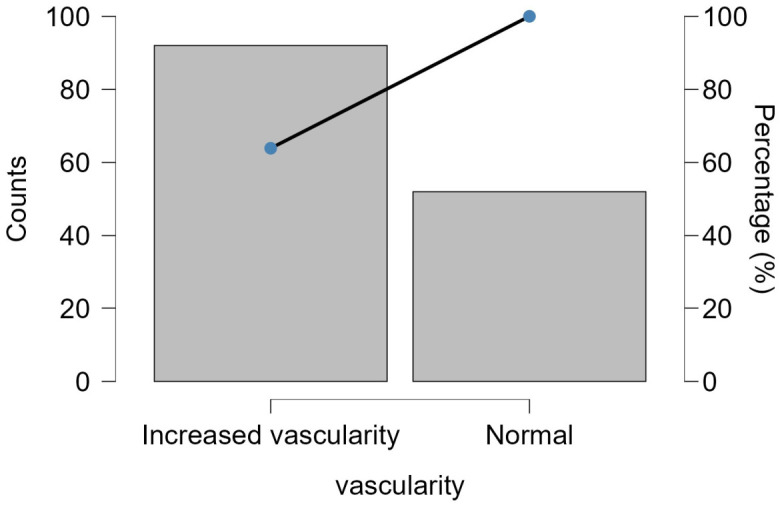
Distribution of Doppler vascularity patterns in thyroid lesions (n = 144).

Increased vascularity was observed in 63.9% of the cases, which could be due to increased metabolic activity or inflammation. The other lesions had normal vascularity (36.1%), as shown in Figure 1.

**Figure 2:**
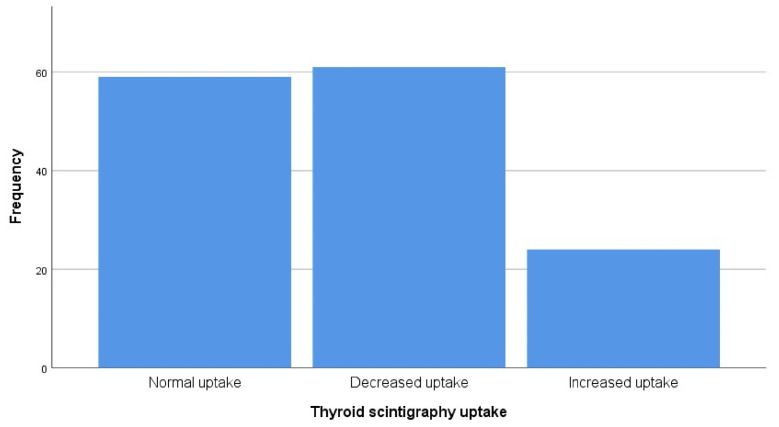
Distribution of thyroid scintigraphy uptake patterns in thyroid lesions (n = 144).

There was variation in scintigraphic uptake in thyroid lesions (normal, decreased, or increased), which reflects thyroid function and lesion type (Figure 2). Notably, 43.1% of the lesions were within the normal range while most thyroid lesions showed increased uptake (55.6%). Such high uptake levels are usually associated with hyperfunctioning portions.

**Figure 3:**
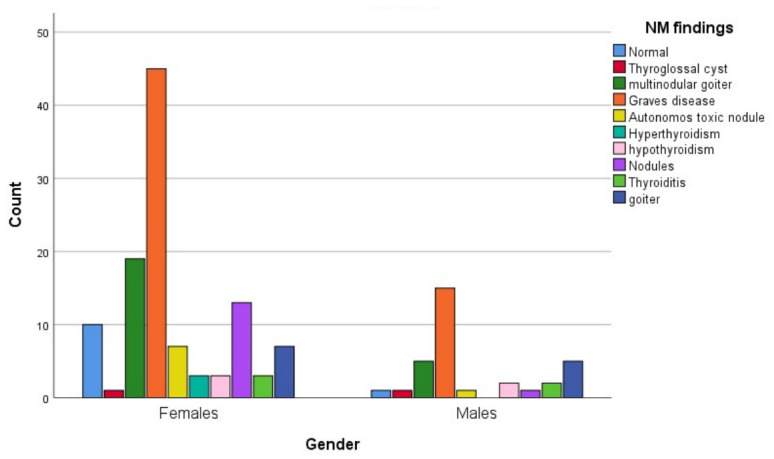
Distribution of nuclear medicine imaging (NM) findings according to gender (n = 144).

The prevalence of thyroid disease was higher in females than in males. The most frequently diagnosed NM lesions were Graves’ disease and multinodular goiters (Figure 3).

**Figure 4:**
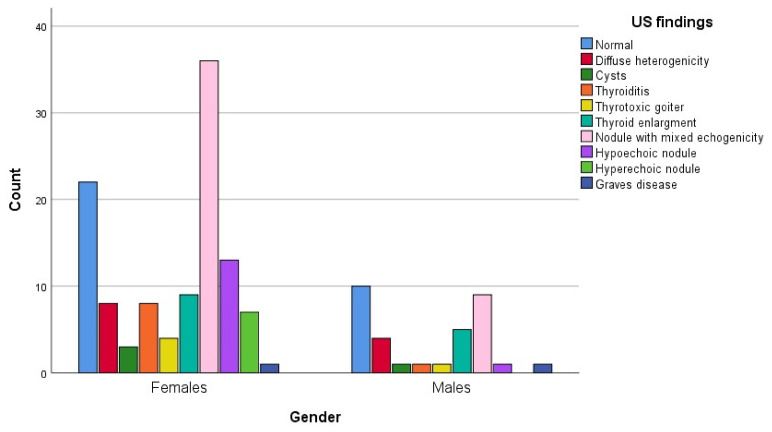
Distribution of ultrasound (US) features of thyroid lesions according to gender (n = 144).

Mixed-echogenicity nodules were more prevalent in females. Normal findings, in contrast, were more common in males. These results indicate notable gender differences in the presentation of thyroid lesions (Figure 4).

**Figure 5:**
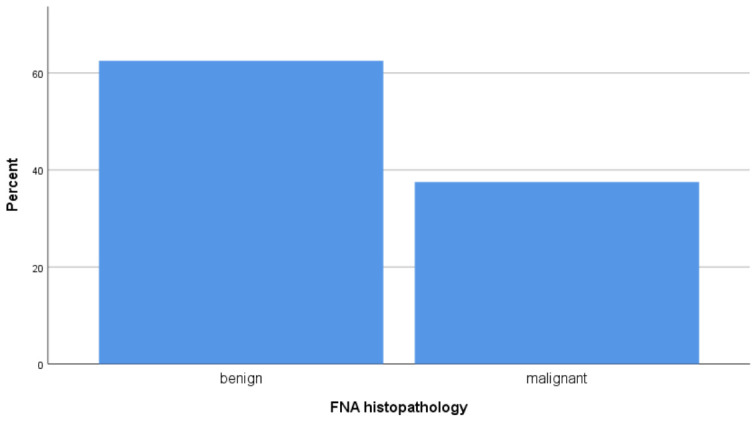
Cytological classification of thyroid lesions based on fine-needle aspiration cytology (FNAC).

Among the nodules subjected to FNAC, the majority were benign (colloid and benign follicular nodules), while papillary and follicular carcinoma comprised the principal malignant subset, together accounting for approximately 37.5% of cases; the remaining cases were indeterminate or inflammatory (Figure 5).

**Figure 6:**
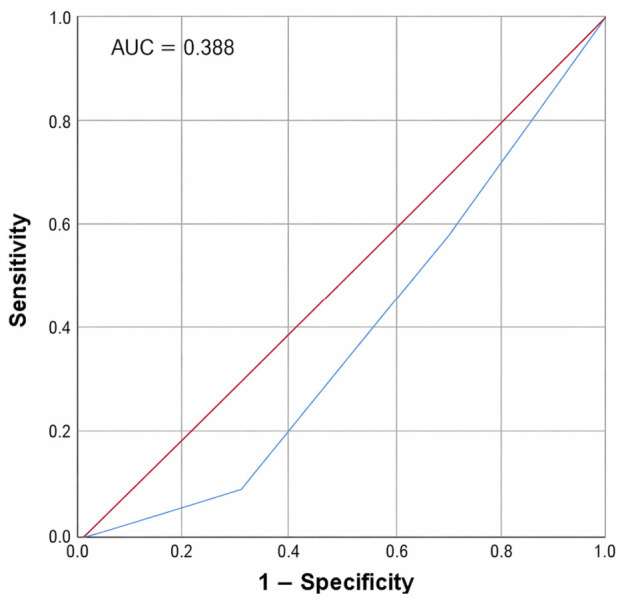
ROC curve for the sensitivity of thyroid scintigraphy uptake in differentiating thyroid malignancy compared to FNAC.

The diagnostic performance of scintigraphy is shown in Figure 6. The ROC curve demonstrates the diagnostic performance of decreased radionuclide (cold nodule) uptake on Tc-99m thyroid scintigraphy in differentiating malignant from benign thyroid nodules, which was compared with FNAC as the gold standard. The model produced an AUC of 0.388 (95% CI: 0.290–0.486), indicating low performance. The sensitivity and specificity at the optimal cut-off were 37.0% and 54.4%, respectively, highlighting the limited efficacy of scintigraphic uptake as a standalone predictor of malignancy and underscoring the need for a more integrated diagnostic model.

**Figure 7:**
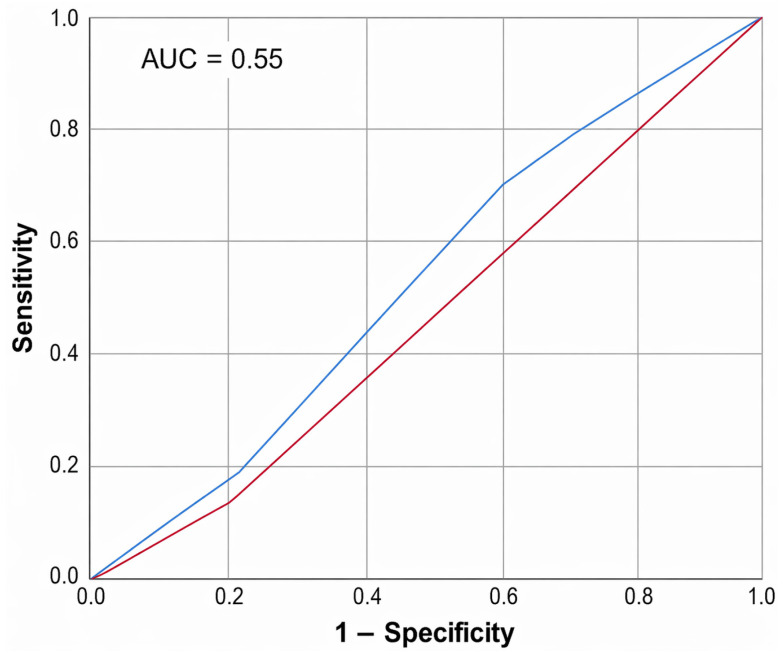
ROC curve for the sensitivity of Doppler vascularity in differentiating thyroid malignancy compared to FNAC.

The diagnostic capability of increased Doppler vascularity in detecting malignant thyroid nodules, which is compared with FNAC as the diagnostic gold standard, is shown in Figure 7. The ROC curve revealed an AUC of 0.514, indicating moderate performance in distinguishing between true positives and false positives. At the optimal threshold, the sensitivity was 70.4% and specificity was 40.0%, indicating that Doppler vascularity provides higher sensitivity but lower specificity when used alone in malignancy risk evaluation.

**Figure 8:**
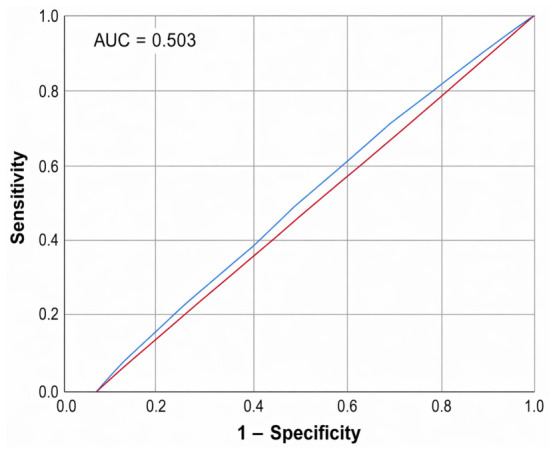
ROC curve for thyroid hormone levels’ sensitivity in detecting thyroid malignancy compared to FNAC.

The diagnostic performance of serum T4 levels in distinguishing between malignant and benign thyroid nodules, which is compared to FNAC as the gold standard, is shown in Figure 8. It presented an AUC of 0.503 with 57.4% sensitivity and 45.6% specificity at the optimal cut-off, indicating that thyroxine level alone has a poor malignancy discrimination ability, underscoring the need for a more integrated diagnostic model.

**Figure 9:**
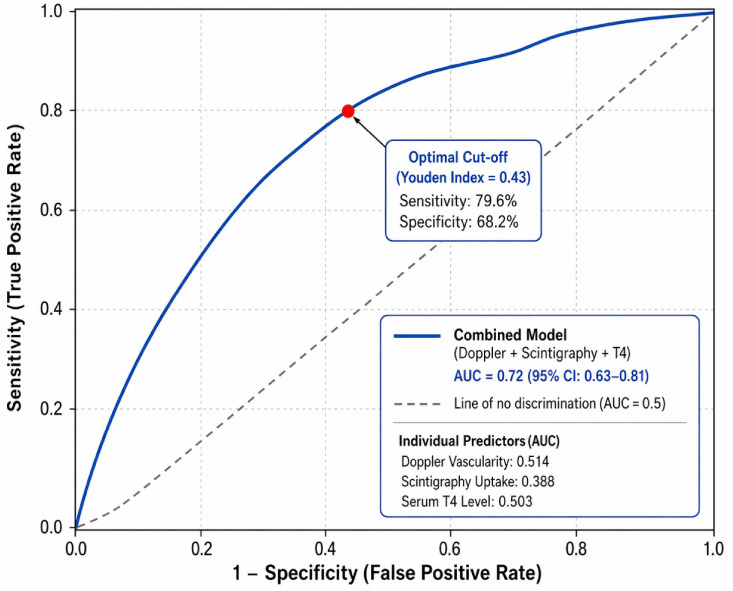
ROC curve for the combined multivariate model including Doppler vascularity, scintigraphy uptake, and serum thyroxine level for predicting thyroid malignancy. The dashed diagonal line represents the line of no discrimination (AUC = 0.5), indicating random classification performance.

Next, we tested an integrated model combining Doppler vascularity, Tc-99m scintigraphy uptake pattern, and serum thyroxine (T4) level. The combined model achieved good diagnostic performance (AUC = 0.72, 95% CI: 0.63–0.81), outperforming the individual imaging approaches, with the optimal cutoff yielding 79.6% sensitivity and 68.2% specificity (Figure 9).

## 4. Discussion

This study attempted to provide a detailed characterization of thyroid gland abnormalities based on results obtained from different imaging modalities, observation of demographic details, the appearance of lesions, and any possible correlation between imaging results and functional and pathological aspects of the thyroid gland. Doppler ultrasound, thyroid scintigraphy with Tc-99m, and serum thyroxine concentrations were combined to characterize thyroid lesions. This multi-imaging approach allows for structural and functional evaluations of thyroid disease, thereby improving the accuracy in differentiating benign from malignant lesions [16,17], improving diagnostic accuracy and enabling tailored treatment. This study found that women and aging populations were the most predisposed to thyroid disorders, with females more likely to experience thyroid abnormalities like mixed-echogenicity nodules and goiters.

The current study adds to the existing literature by considering the holistic interrelationship of the vascular, functional, and biochemical components of the thyroid, rather than the disaggregated approach of considering imaging modalities in isolation. While previous studies have described the diagnostic functions of either Doppler ultrasound or scintigraphy, there is little research on integrating these modalities, along with serum thyroxine levels, within a single framework. The main findings of this study demonstrate significant relationships between Doppler vascularity, scintigraphic uptake, and thyroxine levels, and underscore the need for a multilayered, dimensional approach to diagnostics. This is clinically relevant in the context of paradoxical findings, where a single diagnostic tool is insufficient to interpret them.

The results of this study agree with those of previous studies that studied the demographic patterns of thyroid disorders and found higher rates among females. Fang et al. found that the prevalence of thyroid disease was greater in women than in men in all age groups, except for the 70 years and above group [18]. Consistently, studies have shown that thyroid dysfunction is more common in women than in men. Several other studies have also indicated that age and sex are important determinants of the prevalence and manifestation of thyroid diseases. Other studies have also showed a female predominance, especially in the reproductive or post-reproductive periods [19,20].

Doppler ultrasound is commonly used to evaluate vascularity, and can identify the blood flow patterns in different thyroid diseases. This study found that Doppler sensitivity for characterizing hypervascular thyroid malignant nodules was 70.4%. Consistently, Sushmitha [21] and Ebeed et al. [22] reported sensitivities of 74.4% and 79.2% for ultrasound in discriminating malignant thyroid nodules, respectively. Therefore, the 40% specificity, 70.4% sensitivity, and 51.4% accuracy observed in our study indicates that increased vascularity could differentiate between benign and malignant nodules.

There was a significant association between thyroid scintigraphy uptake and Doppler vascularity. Thyroid lesions with increased uptake exhibited hypervascularity. This is particularly relevant because, in general, both increased vascularity and hyperfunctioning thyroid tissue are connected, as shown by the increased uptake of radiotracer in scintigraphy [16,21]. Consistently, a previous study reported that color Doppler sonography is an effective technique for identifying solitary hypervascular thyroid lesions and stratifying patient risk groups.

In settings with a relatively low incidence of malignant thyroid nodules, such as primary or secondary care facilities, a combination approach utilizing ultrasound and thyroid scintigraphy could be employed to identify suspicious nodules for further evaluation via FNAC, particularly solid hyperfunctioning (“cold”) lesions [22]. The combination of these imaging methods provided insights into thyroid lesions and demonstrated significant correlations between scintigraphy uptake and T4 levels and between scintigraphy uptake and Doppler vascularity (*p*-values of <0.001 and 0.005) [16,21].

This study showed a strong correlation between the echogenicity of thyroid nodules and scintigraphy uptake, which is of practical importance. Mixed-echogenicity nodules were predominantly linked to diminished uptake, whereas hypoechoic nodules frequently exhibited normal uptake. These echogenic patterns indicate the functional condition of thyroid nodules, aiding in differentiating benign from potentially malignant characteristics based on uptake levels. The decreased uptake of mixed-echogenicity nodules could be used in non-invasive screening to determine the functional aspects of thyroid nodules and therefore reduce the reliance on invasive techniques like FNAC [23,24].

Thyroid scintigraphy is essential for identifying functional problems in both nodules and the entire thyroid gland by evaluating total radioactive uptake. Our study revealed that uptake patterns for numerous thyroid isotopes are conclusive for determining pathology. In this study, the sensitivity of scintigraphy uptake was 37% with an AUC of 0.388 for nodules with decreased uptake (cold nodules). A meta-analysis reported a similar AUC of 0.38, with a sensitivity of 0.66 and specificity of 0.36 for the diagnostic performance of radionuclide imaging in characterizing malignant thyroid nodules [25]. Another study reported that 38.5% of hypofunctioning nodules and 2.5% of hyperfunctioning nodules identified by scintigraphy are malignant [26]. In contrast, other studies reported higher sensitivities [16,27,28]. The lower sensitivity in our study could be attributed to the small sample size and the nature of this study as a single-center study. Furthermore, the aim of this study was to create an integrative approach rather than to assess the diagnostic performance.

Although TSH levels are more strongly correlated with the risk of malignancy, T4’s biological role in tumor behavior warranted its inclusion. Thyroid hormones affect cell proliferation, apoptosis, invasion, and the formation of new blood vessels (angiogenesis), with T4 promoting tumor growth via integrin-mediated pathways [29]. This study found a significant association between the levels of thyroxine and thyroid scintigraphy uptake. It was observed that 57.4% of malignant nodules had elevated T4 levels (sensitivity of 57.4%, 95% CI: 43.2–70.8%), but the specificity was modest (45.6%) and the AUC was no better than chance (AUC = 0.503). The NPV was 64.1%, so a normal T4 level indicates a slightly decreased risk of cancer but not no risk. These findings are consistent with previously published studies, which reported that thyroxine alone performs poorly in determining thyroid malignancy [30,31]. On the other hand, T4-elevating conditions are benign (e.g., Graves’ disease or toxic multinodular goiter), explaining the low specificity. These isolated T4 results demonstrate the inadequacy of using T4 level alone to assess malignancy risk. T4 level should be used along with other imaging results, like Doppler vascularity (sensitivity of 70.4%, AUC = 0.514) or scintigraphic uptake patterns (specificity of 54.4%, AUC = 0.388), to provide more clinical context. Integration of biochemical measures with Doppler ultrasound and scintigraphy imaging can improve pre-FNAC triage. This integrative method should be considered for patients with contradictory results (e.g., T4 is elevated but the nodules are not vascular and are cold) so that patients do not have to undergo unnecessary invasive procedures on clearly hyperfunctioning lesions that are likely benign.

Studies have shown that elevated serum thyroxine (T4) levels often correlate with decreased uptake on scintigraphy, particularly in conditions such as hyperthyroidism, where the gland may exhibit low or no uptake despite elevated circulating T4 levels [24,32]. This phenomenon is often noted in patients with subacute thyroiditis, where a transient thyrotoxic state can lead to high serum thyroxine levels while scintigraphy reveals low uptake [24]. Overall, the interplay between thyroxine levels and scintigraphy uptake is complex and necessitates careful consideration of clinical context, medication history, and underlying thyroid pathology to ensure accurate diagnosis and effective management of thyroid disorders [30,33].

Despite the AUC values of the individual modalities indicating limited discriminative ability, the primary goal of this study was to integrate Doppler vascularity, scintigraphy uptake, and thyroxine level rather than improve their standalone diagnostic accuracy. The integrated model showed increased accuracy in diagnosing thyroid malignancy, with a sensitivity of 79.6% and an AUC of 0.72, which is considerably higher than those of the individual approaches. Despite this improved diagnostic performance, the practical utility of the integrated model in clinical practice must be interpreted cautiously. Because of the study’s design and the moderate AUC value, these results are more hypothesis-generating and exploratory than direct findings usable for clinical decision-making, and the proposed multimodal framework will need to be prospectively validated in larger, more precisely defined cohorts to be integrated into direct clinical use. In the multivariate regression model, the only independent predictor of malignancy was increased Doppler vascularity. The addition of scintigraphy and thyroxine improved the model’s overall performance. Giovanella et al. listed several different diagnostic techniques available in clinical thyroidology and suggested their logical integration [12]. Importantly, this study contributes to the existing literature by incorporating Doppler vascularity, scintigraphy uptake, and thyroxine hormone into one model. Although previous studies have analyzed these variables, it has usually been performed independently. The uniqueness of the current study is its holistic approach to evaluating the various facets of thyroid lesions. The integration of these parameters provides a unique understanding of the disease process by assessing the vascularity, functionality, and hormonal aspects, which are inadequately addressed when the techniques are evaluated in isolation. Therefore, the multimodal structural–functional–biochemical method is the best approach for risk stratification. This model could be utilized in the diagnosis of thyroid nodules in order to reduce the risk of performing unnecessary invasive procedures in patients.

A critical factor to consider when analyzing the current findings is the study population’s clinical and metabolic characterization, which is clinically sparse. While imaging and biochemical parameters were thoroughly evaluated, various individual patient factors (including comorbidities, body mass index, metabolic profile, medications, and clinical thyroid symptoms) were frequently absent. These can affect thyroid physiology, the patterns of vascularity, and radionuclide uptake, and could explain the variability in diagnostic performance. Therefore, the comprehensive clinical characterization deficit should be considered when integrating multimodal imaging findings.

### Limitations

The major limitation of this study is its retrospective design. Another limitation is that this study was conducted at a single center, limiting the ability to generalize the findings. Because the sample only included 144 patients, the findings may be subject to selection bias and limited statistical power. Another significant limitation is the study population’s heterogeneous composition, which included a considerable number of patients with functional thyroid disorders, such as Graves’ disease and nodular pathologies. While this reflects daily clinical practice, where imaging is conducted for a broad range of thyroid disorders, it may limit the direct relevance of the findings for populations with increased risks of malignancy. Such hyperfunctional conditions may alter diagnostic performance indicators, particularly specificity, and further constrain the generalizability of the findings to populations undergoing hyperplastic evaluations of thyroid nodules. Furthermore, there is a lack of comprehensive clinical and metabolic data, including body mass index, glucose metabolism, and lipid profile, which may affect thyroid characteristics. As a retrospective imaging-based study, these parameters were not consistently available, potentially preventing a comprehensive interpretation of the findings.

Future research could include larger cohorts to enhance the diagnostic confidence. It is well known that the type and frequency of thyroid disease can vary by geography, culture, and demographics. Increasing the number of centers would make the data more heterogeneous and increase the external validity of the findings.

## 5. Conclusions

The integration of color Doppler ultrasound, Tc-99m thyroid scintigraphy, and serum thyroxine measurement could provide physicians with correlational morphological and functional evaluations of thyroid lesions. Despite the limited diagnostic performances of the individual parameters, each with an AUC of approximately 0.5, the combined approach provides additional diagnostic information for the evaluation and management of thyroid lesions, particularly in cases with ambiguous and contradictory imaging results. Overall, this combination should provide greater interpretive value when assessing malignancy. These results collectively endorse the use of a sequential, multimodal algorithm wherein (i) gray-scale sonography delineates morphology, (ii) Doppler vascularity and scintigraphy enhance functional assessment, and (iii) biochemical data clarify discordant cases prior to FNAC or surgical intervention. This kind of approach could improve pre-procedural risk stratification, minimize unnecessary biopsies, and optimize patient management.

## Figures and Tables

**Table 1:** Demographic characteristics of the study population.

Characteristic	Frequency	Percent
Gender		
Female	111	77.1
Male	33	22.9
Age group (years)		
5–20	28	19.4
21–30	15	10.4
31–40	30	20.8
41–50	34	23.6
51–60	26	18.1
61–70	9	6.3
71–80	2	1.4
Total	144	100.0

**Table 2:** Characterization of thyroid lesions using NM.

NM Finding	Frequency	Percent
Normal	11	7.6
Thyroglossal cyst	2	1.4
Multinodular goiter	24	16.7
Graves’ disease	60	41.7
Autonomous toxic nodule	8	5.6
Hyperthyroidism	3	2.1
Hypothyroidism	5	3.5
Nodules	14	9.7
Thyroiditis	5	3.5
Goiter	12	8.3
Total	144	100.0

**Table 3:** Association between thyroxine level and thyroid scintigraphy uptake.

			Thyroid Uptake		
Thyroxine Level		Normal	Decreased	Increased	Total
Normal	Count	17	41	4	62
	% within the normal distribution of thyroxine	27.4%	66.1%	6.5%	100.0%
High	Count	41	19	20	80
	% within the normal distribution of thyroxine	51.2%	23.8%	25.0%	100.0%
Low	Count	1	1	0	2
	% within the normal distribution of thyroxine	50.0%	50.0%	0.0%	100.0%
Total	Count	59	61	24	144
	% within the normal distribution of thyroxine	41.0%	42.4%	16.7%	100.0%
*p*-value	<0.001				

**Table 4:** Association of thyroid scintigraphy uptake with Doppler vascularity in thyroid lesions.

		Doppler Vascularity		
Thyroid Scintigraphy Uptake		Normal	Increased	Total
Normal	Count	22	37	59
	% within normal distribution of thyroid uptake	37.3%	62.7%	100.0%
Decreased	Count	28	33	61
	% within the normal distribution of thyroxine	45.9%	54.1%	100.0%
Increased	Count	2	22	24
	% within normal distribution of thyroid uptake	8.3%	91.7%	100.0%
Total	Count	52	92	144
	% within normal distribution of thyroid uptake	36.1%	63.9%	100.0%
*p*-value	0.005			

**Table 5:** Diagnostic performance of Doppler vascularity, scintigraphy uptake, and thyroxine hormone in characterization of thyroid lesions compared to FNAC.

Parameter	Sensitivity	Specificity	Accuracy	PPV	NPV	95% CI for Test Sensitivity
Doppler vascularity	70.4	40	51.4	41.3	69.2	56.4–82%
Scintigraphy uptake (cold nodules)	37	54.4	47.9	32.8	59	24.3–51.3%
Thyroxine hormone level	57.4	45.6	50	38.8	64.1	43.2–70.8%

**Table 6:** Multivariate logistic regression analysis of Doppler vascularity, scintigraphy uptake, and thyroxine level as predictors of thyroid malignancy.

	B	SE	Wald	*p*-Value	OR	95% CI
Increased Doppler vascularity	0.87	0.37	5.53	0.019	2.39	1.15–4.96
Decreased scintigraphy uptake	0.60	0.33	3.31	0.069	1.82	0.96–3.44
Increased scintigraphy uptake	−0.29	0.45	0.41	0.521	0.75	0.31–1.82
High thyroxine level	0.45	0.32	1.98	0.159	1.57	0.84–2.94
Constant	−1.30	0.43	9.12	0.003	0.27	—

## Data Availability

Data will be available on request.

## Abbreviations

The following abbreviations are used in this manuscript:

CT	Computed Tomography
FNAC	Fine-needle aspiration cytology
NM	Nuclear medicine
PACS	Picture Archiving and Communication System
Tc-99m	Technetium-99m
T4	Thyroxine
ROC	Receiver operating characteristic
AUC	Area under the curve
CI	Confidence interval
PPV	Positive Predictive Value
NPV	Negative Predictive Value
SPSS	Statistical Package for the Social Sciences
IRB	Institutional Review Board
